# Positive Intraoperative Bile Culture and Antibiotic Resistance Increase the Risk of Pancreatic Fistula in Patients After Pancreatoduodenectomy

**DOI:** 10.3390/jcm14020455

**Published:** 2025-01-12

**Authors:** Michael Hoffmann, Lena Anthuber, Matthias Anthuber, David Pinto, Matthias Schrempf

**Affiliations:** Department of General, Visceral and Transplantation Surgery, University Hospital Augsburg, Stenglinstr. 2, 86156 Augsburg, Germany; lena.anthuber@uk-augsburg.de (L.A.); matthias.anthuber@uk-augsburg.de (M.A.); david.pinto@uk-augsburg.de (D.P.); matthias.schrempf@uk-augsburg.de (M.S.)

**Keywords:** bile culture, pancreatic fistula, POPF, pancreatoduodenectomy

## Abstract

**Background/Objectives**: A positive intraoperative bile culture (bacterobilia) is considered to be a risk factor for increased morbidity after pancreatoduodenectomy. The aim of our study was to describe the frequency of bacterobilia with a special emphasis on antibiotic resistance and to analyze the association of these findings with postoperative complications, in particular with postoperative pancreatic fistula. **Methods**: From a prospective database, patients with available intraoperative bile cultures (n = 95) were selected and analyzed. Microbiological test results reported the type of bacteria as well as sensitivity and resistance patterns. Associations between culture results, antibiotic resistance, and postoperative outcomes were assessed. **Results**: Among 95 patients that were included in this trial, 71 (74.7%) had a positive bile culture. A total of 29.6% (21/71) of patients with positive bile cultures developed POPF grade B/C compared to 8.3% (2/24) of patients with negative bile cultures (*p* = 0.052). The difference in CR-POPF became statistically significant when at least one of the isolated microorganisms was resistant to ampicillin/sulbactam, the perioperative antibiotic administered for prophylaxis. CR-POPF was diagnosed in 38.5% (15/39) of patients with antibiotic resistance vs. 14.3% (8/56) of patients without resistant microorganisms (*p* = 0.007). We also identified the isolation of *Enterococcus* spp. (*p* = 0.006), resistant *Enterobacter* (*p* = 0.031), or resistant *Escherichia coli* (*p* = 0.027) as risk factors for pancreatic fistula. **Conclusions**: The isolation of antibiotic-resistant strains in a positive bile culture is a major risk factor for the development of pancreatic fistula after pancreatoduodenectomy. The most relevant bacteria in our study were *Enterococcus* spp., *Enterobacter cloacae*, and *Escherichia coli*. Thus, broad-spectrum antimicrobial prophylaxis with efficacy against these microorganisms and with low resistance rates should be routinely administered perioperatively.

## 1. Introduction

Pancreatoduodenectomy (PD) is a frequently performed surgical procedure for malignant or benign diseases of the pancreatic head, the distal common bile duct, the ampullary region, or the duodenum. In high-volume centers, it has become a routine and safe procedure with low mortality. Despite this progress, major pancreatic resections are still associated with significant morbidity [1,2]. One of the most serious complications after PD is postoperative pancreatic fistula (POPF). The International Study Group of Pancreatic Fistula (ISGPF) grades POPF B and C as clinically relevant (CR-POPF) [3]. CR-POPF increases the length of hospital stay, the reoperation rate, and the risk of postoperative mortality and is also associated with delay or omission of adjuvant chemotherapy, thus compromising oncological outcome [4,5].

There is an ongoing debate about the role of positive intraoperative bile cultures and preoperative stenting. Stenting often leads to bacterial contamination and to a change in the biliary microbiology, including a higher frequency of a polymicrobial spectrum and a shift towards resistant microorganisms [6]. Stents in the biliary tract can lead to biofilm formation, which results in persistent and recurrent bacterial infection and increases the risk of stent occlusion [7]. Cholangitis and previous antibiotic therapy are well-known risk factors for antibiotic resistance. In general, the incidence of antimicrobial resistance is rapidly increasing [8], and hospitalized patients with cancer show a particularly high rate of antibiotic resistance [9]. Recent research also describes changes in the intestinal microbiome as a common phenomenon in patients with pancreatic cancer [10].

Multiple studies [6,11,12,13,14,15,16,17,18,19] and a recent meta-analysis [20] investigated the association between bacterobilia and postoperative complications. An increase in postoperative morbidity is frequently described, and some studies also show an association of bacterobilia with CR-POPF [13,18,19], although the latter is not a consistent finding [20].

As preoperative stenting cannot always be avoided, the role of adequate perioperative antimicrobial prophylaxis has come into focus. Cephalosporins are widely used and recommended by clinical guidelines [21], but in some countries and also in our institution, ampicillin/sulbactam is the preferred choice for antimicrobial prophylaxis due to its superior coverage of *Enterococcus* spp. Clinical studies demonstrate that *Enterococcus faecalis* is one of the most common organisms found at sites of anastomotic leakage. With its ability to produce collagenase, this may be of high clinical relevance [22,23] and is also a possible explanation for the results of a recent randomized controlled trial that showed a reduction in surgical site infections and pancreatic fistula if piperacillin-tazobactam was used instead of cefoxitin as antimicrobial prophylaxis [24].

The objectives of our study were to describe the frequency of positive intraoperative bile cultures with a special focus on antibiotic resistance and to analyze the association between these findings and postoperative complications, especially with CR-POPF.

## 2. Materials and Methods

### 2.1. Trial Design and Participants

This study was conducted as a single-center study at the Department of General, Visceral, and Transplant Surgery at the University Hospital of Augsburg, Germany. Anonymous data were extracted from a prospective database of patients undergoing pylorus-preserving pancreatoduodenectomy that was created as part of a randomized controlled trial, the PORRIDGE study. The study protocol of the randomized study was approved by the ethics committee of the Ludwig-Maximilians-University Munich (reference number 17-605). Informed consent was obtained for participation in the prospective trial and data collection.

**Figure 1:**
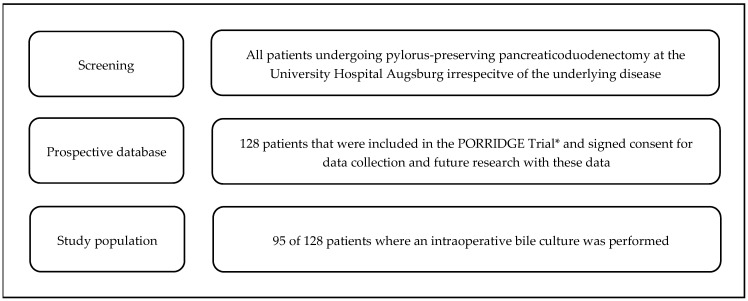
Study Flowchart. * The inclusion criteria and the study protocol for the PORRIDGE trial were published before [25].

In 95 of 128 patients in the database, an intraoperative bile culture was available. These 95 patients served as cohorts for the current study. The study flowchart is shown in Figure 1.

### 2.2. Antibiotic Prophylaxis, Microbiology, and POPF Prophylaxis

Ampicillin/sulbactam was routinely administered as perioperative antibiotic prophylaxis by the anesthesiologist 30 to 60 min before skin incision and repeated every 3 h until the end of the surgical procedure. Intraoperative bile cultures were acquired directly after transection of the common bile duct using a swab. Microbiological test results reported the type of bacteria as well as sensitivity and resistance patterns. All patients received 100 μg of somatostatin subcutaneously intraoperatively before pancreaticojejunostomy creation and 100 μg of somatostatin subcutaneously three times daily thereafter until postoperative day 5, based on the internal standard.

### 2.3. Outcome Parameters

The rate of CR-POPF was assessed according to the ISGPS definition. Complications, comorbidities, operative data, and patient characteristics were collected from the database, including surgical site infections, bile leak, operating time, estimated blood loss, and length of hospital stay. Complications were assessed using the Clavien–Dindo classification and the Comprehensive Complication Index^®^ (CCI) [26,27].

### 2.4. Statistical Analysis

Continuous data are presented as mean ± standard deviation or median with interquartile range, depending on the distribution. Categorical data will be presented as numbers with percentages. Approximately normally distributed continuous variables were compared using the independent *t*-test. Nonnormally distributed continuous variables were compared using the Mann–Whitney U test. Categorical data were compared using the χ2 test. Fisher’s exact test will be used for categorical data if the requirements for the χ2 test are not met. A two-sided *p* < 0.05 was considered significant. Outcome parameters were tested for differences between patients with positive and negative intraoperative bile culture and the presence of specific microorganisms and resistant bacteria in a univariate analysis. A multivariable binary logistic regression analysis was performed for the endpoint CR-POPF to adjust to risk factors, including the variables “resistance to ampicillin” and the patient characteristics of sex, age, BMI, diabetes, and ASA. A ROC curve was calculated to evaluate the fit of the logistic regression model. Statistical analyses were undertaken using SPSS^®^ for macOS^®^, version 31 (IBM, Armonk, NY, USA).

## 3. Results

### 3.1. Study Cohort and Patient Characteristics

The PORRIDGE study was conducted between February 2018 and December 2023. A total of 128 patients with pylorus-preserving pancreatoduodenectomy were included. In 95 of these patients, an intraoperative bile culture was performed. Among these 95 patients, 71 (74.7%) had a positive bile culture, and in 24 (25.3%) the bile was sterile. In 15 (15.7%), 1 microorganism grew, and in 56 (58.9%), the bile colonization was polymicrobial. The baseline characteristics of both groups are shown in Table 1. Patients with positive bile culture were older than those without bacterobilia (70.9 vs. 66.0 years, *p* = 0.01). No other statistically significant differences were observed in baseline characteristics.

### 3.2. Microbiological Analysis and Antibiotic Resistance

The most common species in bile cultures from intraoperative swabs were *Enterococcus* spp., which were isolated in 40% of patients. Common Gram-negative strains were *Klebsiella* spp., *Enterobacter cloacae*, and *Escherichia coli*. Resistance to ampicillin/sulbactam, the perioperative antibiotic administered for prophylaxis, was common. A total of 39 of 95 patients (41%) had at least one bacterial strain with resistance to this regimen. *Enterobacter cloacae* was the most common resistant microorganism (18.9% of patients). Table 2 shows detailed results of bile culture analyses.

### 3.3. Outcome and Complications

Table 3 demonstrates the outcome of patients with positive and negative bile cultures, and Table 4 shows the differences dependent on antibiotic resistance. A total of 29.6% (21/71) of patients with positive bile cultures developed POPF Grade B/C compared to 8.3% (2/24) of patients with negative bile cultures (*p* = 0.052). The difference in CR-POPF became highly statistically significant when at least one of the detected microorganisms was resistant to ampicillin/sulbactam, the antibiotic routinely administered for perioperative prophylaxis. CR-POPF was diagnosed in 38.5% (15/39) of patients with antibiotic resistance compared to 14.3% (8/56) of patients without detection of resistant microorganisms (*p* = 0.007).

Surgical site infections were found in 11.3% of patients with and in 4.2% of patients without bacterobilia (*p* = 0.44). It occurred in 15.4% with resistance to ampicillin/sulbactam and in 5.4% without resistance (*p* = 0.15).

We found no statistically significant differences in any other outcome parameters.

### 3.4. Association of Specific Microorganisms and Postoperative Outcome

As mentioned, resistance to ampicillin/sulbactam was a significant risk factor for the development of CR-POPF (*p* = 0.009). As Table 5 shows, the presence of specific microorganisms was also associated with a higher incidence of CR-POPF. If *Enterococcus* spp. were found in bile cultures, this was a risk factor for CR-POPF, independent of antibiotic resistance (*p* = 0.006). *Escherichia coli* and *Enterobacter cloacae* also lead to an increased risk of CR-POPF. This association became even clearer if those species were resistant to ampicillin/sulbactam (*p* = 0.027 for *Escherichia coli* and *p* = 0.031 for *Enterobacter cloacae*). For other microorganisms, we found no association with CR-POPF.

To further investigate the relationship between the detection of an ampicillin-resistant bacterium and the occurrence of CR-POPF, we performed a multivariate analysis, adjusting for the potential risk factors: sex, BMI, age, diabetes, and ASA score (Supplementary Table S1). Resistance to ampicillin/sulbactam was associated with increased risk for CR-POPF in the multivariable analysis (OR 4.00; 95% CI 1.26–12.75; *p* = 0.019). Other risk factors for CR-POPF were male sex (OR 5.32; 95% CI 1.48–19.10; *p* = 0.010) and BMI ≥ 30 kg/m^2^ (OR 5.88; 95% CI 1.24–28.03; *p* = 0.026). The ROC curve calculated to evaluate the logistic regression model did show a good fit of the model (Supplementary Figure S1).

## 4. Discussion

Our study demonstrates the importance of positive intraoperative bile culture after pancreatoduodenectomy. It shows that the presence of antibiotic-resistant bacteria is highly relevant with regard to postoperative complications, especially CR-POPF. Of 95 patients included in our study, 74.7% had a positive bile culture, and 41% had at least one bacterial strain with resistance to ampicillin/sulbactam, the antimicrobial prophylaxis administered for prophylaxis. We found a trend towards a higher incidence of POPF Grade B/C from 8.3% of patients with negative bile cultures to 29.6% of patients with positive bile cultures (*p* = 0.052). The difference became statistically significant if at least one of the detected microorganisms was resistant to ampicillin/sulbactam (*p* = 0.007).

As mentioned above, the association of positive bile cultures and postoperative complications after PD has been described before, but study results are inconsistent. A meta-analysis from 2023, including eight articles, only showed an association with surgical site infections but could not demonstrate a higher incidence of POPF [20]. However, a recently published multicenter, randomized controlled trial with 778 participants could clearly establish an association between positive bile culture and CR-POPF when cefoxitin was administered as perioperative antimicrobial prophylaxis. Cefoxitin proved to be an insufficient prophylaxis with a high incidence of resistant strains. When piperacillin/tazobactam, which has much lower resistance rates, was administered instead, the incidence of CR-POPF was reduced from 19.0% to 12.7% [13,24]. Piperacillin/tazobactam also reduced the incidence of surgical site infections and even 30-day mortality. These findings might at least partially explain the different study results on this topic. Most studies in the past focused on positive bile cultures per se but not on antibiotic resistance. Depending on local resistance patterns and the administered prophylaxis, the incidence of CR-POPF might be lower and the association with bacterobilia less clear.

Of note, Pretzsch et al. found a higher rate of POPF, wound infections, and a higher mean Comprehensive Complication Index in patients with bacterobilia, but some of these effects were offset by the administration of adequate perioperative antibiotic prophylaxis [19].

A retrospective study in a population with a high risk for pancreatic fistula showed a reduced rate of CR-POPF and a shorter length of hospital stay if perioperative therapy was extended towards broader-spectrum antibiotics and longer application [28].

Sourouille et al. examined 175 patients after pancreatoduodenectomy and compared infectious complications in high-risk and low-risk patients [29]. The high-risk group received a 5-day course of perioperative antimicrobial therapy secondarily adapted to the bile antibiogram. The low-risk group received only the usual antimicrobial prophylaxis. Despite more positive bile cultures, the high-risk group had a reduced rate of infectious complications, presumably as a result of this active prevention strategy. The initial regimen was found to be inappropriate in the majority of patients with infectious complications.

Our study results fit very well into this context. Since at least one resistant microorganism was isolated in 41% of patients, ampicillin/sulbactam was suboptimal as perioperative prophylaxis. The incidence of CR-POPF was more than twice as high in patients with resistant strains compared to patients without resistant strains (*p* = 0.007), which is clinically meaningful. Surgical site infections occurred in 11.3% of patients with and 4.2% of patients without bacterobilia, but we were unable to demonstrate a statistically significant difference in this complication. The latter is most likely explained by the small study population and low numbers of SSIs.

The specific microorganisms that were isolated in bile cultures are an important issue. The human gut microbiome is discussed as a key driver of wound and anastomotic complications [30]. Recently, its role in pancreatic diseases has also come into focus, and the concept of a “microbiota–pancreas axis” has been introduced [10]. It explicates a bidirectional communication system that describes how the gut microbiota can influence the pancreas and, conversely, how pancreatic disorders can affect the intestinal microbiome.

Enterococci are among the best-studied species. They are described as collagenolytic and can activate additional enzymatic pathways that lyse connective tissue [22,23,30,31]. In our study, the isolation of *Enterococcus* spp. was associated with a higher incidence of CR-POPF. This supports the recently published results by Ellis et al. [13]. In addition, Kimura and colleagues collected fluid from the drain placed at the pancreaticojejunostomy on postoperative days 1, 3, and 6. In their study, *Enterococcus* spp. were more frequently isolated in patients with CR-POPF than in patients without pancreatic fistula [18].

The presence of *Escherichia coli* and *Enterobacter cloacae* in bile cultures was a risk factor for CR-POPF in our study, especially when these microorganisms were resistant to the prophylaxis administered (*p* = 0.027 for *Escherichia coli* and *p* = 0.031 for *Enterobacter cloacae*). Heckler and colleagues found the same association between *Escherichia coli* and CR-POPF [14], and the association of *Enterobacter cloacae* and pancreatic fistula has also been described previously [13]. These findings could be of therapeutic relevance and emphasize the concept that not all bacteria have the same influence on postoperative complications. In theory, some might even have a protective effect [14].

As mentioned, preoperative biliary drainage and stenting are well-known risk factors for bacterial contamination of the biliary tract. These interventions change the biliary microbiome towards a more polymicrobial spectrum and more resistant species [6]. In addition, biliary obstruction per se can lead to bacterial overgrowth and subsequent colonization of the bile duct [32]. Disruption of the antibacterial barrier by cholestasis and increased pressure in the biliary system facilitate bacteria migration from the duodenum into the biliary tract [33]. Of note, Goel et al. examined the influence of neoadjuvant chemotherapy on the biliary microbiome in pancreatic cancer [34]. Neoadjuvant therapy led to a significant increase in cephalosporin resistance and a higher rate of Enterococci, Enterobacter, and *Klebsiella* in bile cultures. Immunosuppression and prolonged periods of stenting with cholangitis may be contributing to this result.

A plausible conclusion from our study is the importance of broad-spectrum antimicrobial prophylaxis with efficacy against *Enterococcus* spp. and common Gram-negative species, particularly *Enterobacter cloacae* and *Escherichia coli*. In our study, 13.7% of patients had at least one species with resistance to piperacillin/tazobactam compared to 41% with at least one resistance to ampicillin/sulbactam. This underlines the findings of D’Angelica et al. in their recent randomized trial [24], which were confirmed in a meta-analysis by Kumar and colleagues [35]. Both articles conclude that piperacillin/tazobactam as antimicrobial prophylaxis lowers the risk of postoperative complications after PD.

To date, there is less evidence on the duration of perioperative antimicrobial prophylaxis. A meta-analysis from 2023 addressed this question and found a lower incidence of organ/space surgical site infections in patients with preoperative stenting after prolonged antibiotic prophylaxis of more than 24 h [36]. However, most of the included studies were retrospective, and no study examined piperacillin/tazobactam in the intervention and the control group. Thus, the optimal duration of antibiotic prophylaxis remains an open question that should be investigated in a randomized trial. A differentiated approach based on the individual risk for positive bile culture and antibiotic resistance could also be a reasonable strategy and should be further examined.

## 5. Strengths and Limitations

Our study has strengths and limitations. It is one of the few studies that correlate postoperative outcomes with antibiotic resistance in the bile culture. The study cohort is relatively homogenous with a standardized operative procedure. The data were prospectively collected, but this study is of a retrospective nature, a possible source of interpretation bias. It is a single-center trial, which can lead to an overestimation of therapeutic effects [37]. In addition, local resistance patterns may vary between hospitals and countries. At the time of the study, intraoperative bile culture was not performed routinely in all patients at our institution but only at the discretion of the surgeon. For this reason, 33 patients from the original cohort of 128 patients had to be excluded. The cohort is relatively small, and our results need confirmation in additional and larger studies.

## 6. Conclusions

The presence of antibiotic-resistant strains in a positive bile culture is a major risk factor for the development of pancreatic fistula after pancreatoduodenectomy. The most relevant bacteria in our study were *Enterococcus* spp., *Enterobacter cloacae*, and *Escherichia coli*. In view of the current literature, broad-spectrum antimicrobial prophylaxis, such as piperacillin/tazobactam with efficacy against these microorganisms and low resistance rates, should be routinely administered during pancreatoduodenectomy.

## Figures and Tables

**Table 1:** Baseline Characteristics.

	All n = 95	Positive Bile Culture n = 71	Negative Bile Culture n = 24	*p*
Sex, n (%)				
Female	40 (42.1)	27 (38.0)	13 (54.2)	
Male	55 (57.9)	44 (62.0)	11 (45.8)	0.17
Age	69.7 (±8.0)	70.9 (7.7)	66.0 (7.6)	**0.01**
BMI ≥ 30 kg/m^2^, n (%)	12 (12.6)	8 (11.3)	4 (16.7)	0.72
Chronic renal insufficiency, n (%)	5 (5.3)	3 (4.2)	2 (8.3)	0.60
Diabetes mellitus, n (%)	19 (20.0)	11 (15.5)	8 (33.3)	0.08
Weight loss (≥10% within 6 months)	30 (31.6)	24 (33.8)	6 (25.0)	0.42
Neoadjuvant therapy	4 (4.2)	4 (5.6)	0	1.0
ASA III or higher, n (%)	58 (61.1)	45 (63.4)	13 (54.2)	0.42
Portal Vein/Superior Mesenteric Vein involvement	5 (5.3)	3 (4.2)	2 (8.3)	0.60
Malignant histology	79 (83.2)	62 (87.3)	17 (70.8)	0.11

Bold: Statistically significant.

**Table 2:** Microbiological analysis of positive bile cultures.

Microorganism	Prevalence (n = 95 Patients)	Ampicillin/Sulbactam Resistance (n = 95 Patients)
Gram positive		
*Enterococcus* spp.	38 (40.0%)	7 (7.4%)
*Streptococcus* spp.	20 (21.0%)	0 (0.0%)
*Staphylococcus aureus*	2 (2.1%)	0 (0.0%)
*S. epidermidis*	2 (2.1%)	2 (2.1%)
Gram negative		
*Klebsiella* spp.	30 (31.6%)	8 (8.4%)
*Enterobacter cloacae*	20 (21.0%)	18 (18.9%)
*E. coli*	20 (21.0%)	6 (6.3%)
*Citrobacter* spp.	8 (8.4%)	6 (6.3%)
*Proteus vulgaris*	6 (6.3%)	0 (0.0%)
*Hafnia alvei*	4 (4.2%)	4 (4.2%)
*Serratia marcescens*	2 (2.1%)	1 (1.1%)
Other	9 (9.5%)	5 (5.3%)
Anaerobic spec	8 (8.4%)	-
*Candida* spp.	5 (5.3%)	-

**Table 3:** Association of bile culture results and postoperative complications.

	Positive Bile Culture (n = 71)	Negative Bile Culture (n = 24)	*p*
Surgical site infection	8 (11.3%)	1 (4.2%)	0.44
Complication Clavien–Dindo ≥ 3	30 (42.3%)	9 (37.5%)	0.81
Comprehensive Complication Index^®^ (range 0–100)	33.2 (22.6–46.8)	34.6 (21.3–44.1)	0.95
POPF B/C	21 (29.6%)	2 (8.3%)	0.052
Bile leak	7 (9.9%)	3 (12.3%)	1.0
Intraabdominal complication	29 (40.5%)	9 (37.5%)	0.77
Length of hospital stay	16 (12–21)	16.5 (12–21)	0.81

Data are n (%) or median (OR).

**Table 4:** Association of antibiotic resistance and postoperative complications.

	At Least One Microorganism Resistant to Ampicillin/Sulbactam (n = 39)	No Resistance to Ampicillin/Sulbactam (n = 56)	*p*
Surgical site infection	6 (15.4%)	3 (5.4%)	0.15
Complication Clavien–Dindo ≥ 3	17 (43.6%)	22 (39.3%)	0.68
Comprehensive Complication Index^®^ (range 0–100)	33.2 (22.6–46.8)	33.3 (20.9–45.0)	0.79
POPF B/C	15 (38.5%)	8 (14.3%)	**0.007**
Bile leak	4 (10.3%)	6 (10.7%)	1.0
Intraabdominal complication	17 (43.6%)	21 (37.5%)	0.55
Length of hospital stay	16.8 (12–21)	15.9 (12–21)	0.71

Data are n (%) or median (OR). Bold: Statistically significant.

**Table 5:** Risk factors for POPF B/C.

	CR-POPF n = 23	No CR-POPF n = 72	*p*	OR (95% CI)
Positive bile culture	21 (91,3%)	50 (69,4%)	0.052	4.62 (0.996–21.44)
Resistance to ampicillin/sulbactam	14 (60,8%)	25 (34,7%)	**0.009**	3.75 (1.40–10.07)
Resistance to piperacillin/tazobactam	6 (26.1%)	7 (9.7%)	0.055	3.28 (0.97–11.04)
*Enterococcus* spp.	15 (65.2%)	23 (31.9%)	**0.006**	4.00 (1.48–10.76)
*Enterococcus* spp. with resistance to ampicillin/sulbactam	3 (13.0%)	4 (5.6%)	0.25	2.55 (0.53–12.35)
*Klebsiella* spp.	10 (43.5%)	20 (27.8%)	0.16	2.00 (0.76–5.29)
*Klebsiella* spp. with resistance to ampicillin/sulbactam	2 (8.7%)	6 (8.3%)	0.96	1.05 (0.20–5.59)
*Escherichia coli*	8 (34.8%)	12 (16.7%)	0.07	2.67 (0.93–7.69)
*Escherichia coli* with resistance to ampicillin/sulbactam	4 (17.4%)	2 (2.8%)	**0.027**	7.37 (1.25–43.32)
*Enterobacter cloacae*	8 (34.8%)	12 (16.7%)	**0.047**	2.96 (1.01–8.64)
*Enterobacter cloacae* with resistance to ampicillin/sulbactam	8 (34.8%)	10 (13.9%)	**0.031**	3.31 (1.12–9.81)
*Streptococcus* spp.	4 (17.4%)	16 (22.2%)	0.62	0.74 (0.22–2.48)

Bold: Statistically significant.

## Data Availability

The datasets generated and analyzed in the present study are available upon reasonable request to the corresponding author.

## Supplementary Materials

The following supporting information can be downloaded at: https://www.mdpi.com/article/10.3390/jcm14020455/s1, Figure S1: ROC curve; Table S1: Risk factors for CR-POPF—multivariable analysis.
