# Supplementary material for: Positive Intraoperative Bile Culture and Antibiotic Resistance Increase the Risk of Pancreatic Fistula in Patients After Pancreatoduodenectomy
Source: J Clin Med. 2025 Jan 12;14(2):455. doi: 10.3390/jcm14020455 (PMC11766183; doi:10.3390/jcm14020455)
Supplement: Supplementary file 1 [file jcm-14-00455-s001.zip › jcm-3379929-supplementary.pdf]

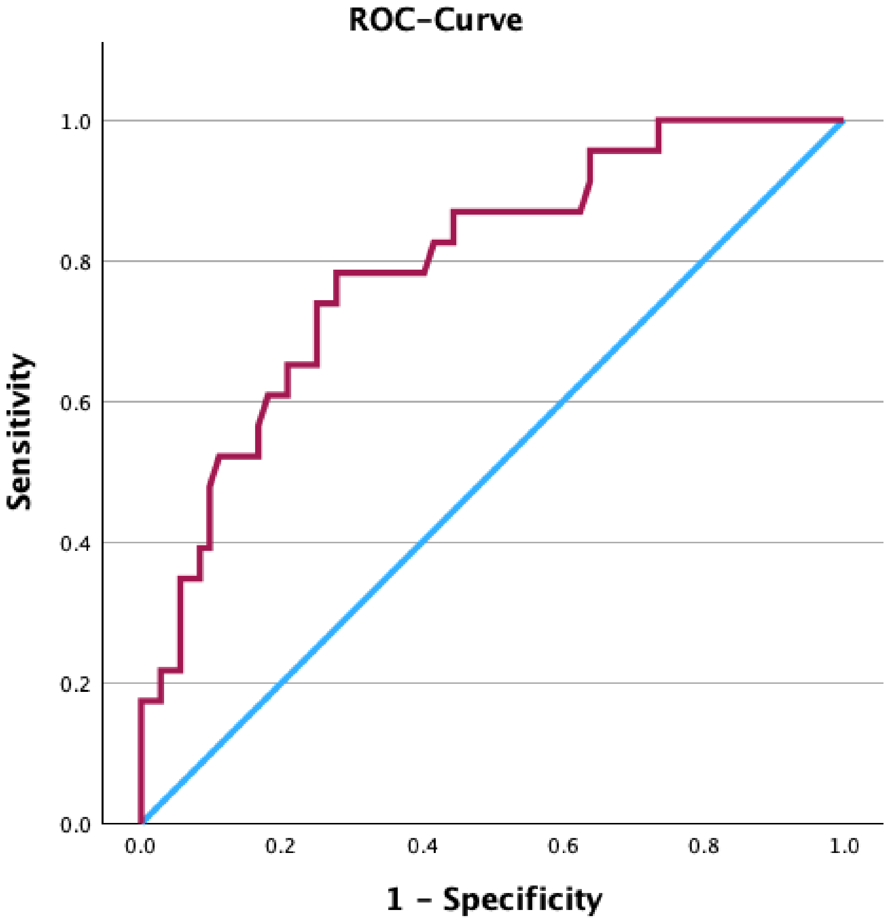

Supplementary Figure S1. ROC Curve. **Area Under the Curve:**Test Result Variable(s): Predicted probability

Area	Std. Error [a]	Asymptotic Sig.[b]	Asymptotic 95% Confidence Interval	
			Lower Bound	Upper Bound
**0.793**	**0.053**	<0.0001	0.690	0.896

a. Under the nonparametric assumption. b. Null hypothesis: true area = 0.5; sig. = significance.

Supplementary Table S1. Risk factors for CR-POPF – multivariable analysis.

	OR	95% CI	*P* (Multivariable)
Resistance to ampicillin/sulbactam	4.00	1.26 – 12.75	**0.019**
Age	1.05	0.97 – 1.13	0.25
Male sex	5.32	1.48 – 19.10	**0.010**
BMI ≥30 kg/m^2	5.88	1.24 – 28.03	**0.026**
Diabetes	1.73	0.39 – 7.67	0.47
ASA score ≥ 3	0.40	0.11 – 1.41	0.16

BMI: body mass index, ASA: American Society of Anaesthesiologists,.
